# Prevention through policy: Urban macroplastic leakages to the marine environment during extreme rainfall events

**DOI:** 10.1016/j.marpolbul.2017.07.024

**Published:** 2017-11-15

**Authors:** Charles Axelsson, Erik van Sebille

**Affiliations:** a Centre for Environmental Policy, Imperial College London, London, UK; b Grantham Institute and Department of Physics, Imperial College London, London, UK; c Institute for Marine and Atmospheric research Utrecht, Utrecht University, Utrecht, Netherlands

**Keywords:** Marine plastic, Urban policy, Coastal environments

## Abstract

The leakage of large plastic litter (macroplastics) into the ocean is a major environmental problem. A significant fraction of this leakage originates from coastal cities, particularly during extreme rainfall events. As coastal cities continue to grow, finding ways to reduce this macroplastic leakage is extremely pertinent. Here, we explore why and how coastal cities can reduce macroplastic leakages during extreme rainfall events. Using nine global cities as a basis, we establish that while cities actively create policies that reduce plastic leakages, more needs to be done. Nonetheless, these policies are economically, socially and environmentally cobeneficial to the city environment. While the lack of political engagement and economic concerns limit these policies, lacking social motivation and engagement is the largest limitation towards implementing policy. We recommend cities to incentivize citizen and municipal engagement with responsible usage of plastics, cleaning the environment and preparing for future extreme rainfall events.

## 1 Introduction

Plastic is increasingly prevalent in the world's oceans. An exact estimate for the amount of plastic in the ocean is unknown, but the amount floating at the surface of the ocean is at least 93 thousand tons (van Sebille et al., 2015). However, estimates for the amount of plastic waste entering the ocean are significantly higher. Jambeck et al. (2015) estimate that coastal countries produce 275 million tons of plastic waste annually, releasing 4.8–12.7 million tons into the ocean, while the Ellen Macarthur Foundation (2016) estimates that of the 78 million tons of plastic packaging produced, 32% leaks into the environment, (roughly 25 million tons) some of which ends in the ocean. A significant fraction of this plastic could be on coastlines. In 2015, the International Coastal Cleanup programme collected nearly 8.2 thousand metric tons of trash on over 40,000 km of coastline globally (ICC, 2016).

This ocean plastic is harmful to marine life, through deformation, maiming, suffocation and death (e.g. Derraik, 2002, Gregory, 2009, Rochman et al., 2015). Furthermore, plastic can help spread invasive species and release toxic chemicals into the environment (Thompson et al., 2009, Kwon et al., 2015, Zettler et al., 2013;). Because biological diversity and species abundance tends to be highest near coastlines, plastic does most harm there (Wilcox et al., 2015, Schuyler et al., 2016, Sherman and van Sebille, 2016), often close to where the plastic enters the ocean.

Marine plastic not only harms marine life, but also impacts our ability to interact with the coast. Potential decreases in marine biota would directly affect the fisheries economy. The tourism industry is also influenced by the presence of coastal plastic on beaches. Ballance et al. (2000) estimate that a loss in the standards of cleanliness on the beaches in Cape Town would result in a 97% loss in the value of those beaches. In Geoje Island, Korea, following an extreme rainfall event in 2011 marine debris cost the island US$29–37 million in tourism losses (Jang et al., 2014). Marine macro-plastics are unsightly and detract from the inherent value of the world's coastlines.

The shoreline does not act as an unlimited plastic sink. Plastic may degrade on the beaches due to chemical and physical erosion and be washed back into the oceans (Cooper and Corcoran, 2010, Corcoran et al., 2009). Plastic found on the shore is seasonal in its input. During rainier seasons, more plastic debris is flushed from land to the sea (Silva-Cavalcanti et al., 2009, de Araujo and Costa, 2006). While the rainy season is more prevalent in the tropics, extreme rainfall events occur across the globe.

Human behavior and policies on land affect the amount of plastic in the oceans. Littering and poor waste management, stormwater discharge, and extreme events such as floods and landslides are large terrestrial sources of marine debris (NOAA, 2016). From inland, the debris travels to the oceans via wind and water, while human activity on the coast creates direct inputs of marine debris. In 2002, 58% of the debris collected in the International Coastal Cleanup was attributed to shore-line and recreational activities (Allsopp et al., 2006).

In cities, street litter is not only unsightly but also expensive to cleanup. England spends £1 billion annually to clean up the 30 million tons of litter generated (Keep Britain Tidy, 2013) and the USA spends upwards of $11 billion annually to clean up litter (NC DPS, 2016). Yet, litter persists; upwards of four trillion cigarette butts, which are frequently made of the plastic cellulose acetate, are improperly disposed of globally every year (ASH, 2015).

Globally, better waste management is a focal point in reducing plastic in the environment. However, there are instances when day-to-day waste management will not suffice in stopping plastic leaking into the ocean. For example, in the immediate aftermath of a tropical storm, resource management is focused on human health, toxic spills and air quality (Institute of Medicine, 2007) as opposed to waste management. Yet, debris including plastic flushes into the ocean during these extreme rainfall events. There is a gap in our understanding of debris and plastics management in the coast and ocean immediately following disaster and extreme rainfall events.

As urban environments grow, stormwater management will become more important. In addition, it is projected that extreme rainfall events will increase due to climate change (Potsdam Institute for Climate Impact Research (PIK), 2015, Intergovernmental Panel on Climate Change (IPCC), 2013, U.S. Climate Change Science Program, 2008). An increase in extreme rainfall events is likely to stress our current stormwater management systems and thus there is an increased potential for plastic to spill into the ocean. Extreme rainfall events only represent a portion of plastic that leaks into the marine environment, however, their growing frequency offers an opportunity to make infrastructure and economic investments to prepare for these events. In addition to removing plastic from the ocean, reducing urban plastics can be co-beneficial for the overall urban environment. Therefore, this study asks the following question: “*Why and how could coastal cities reduce urban plastic leakages due to extreme rainfall events?*”

This study aims to establish how coastal cities reduce plastic leakages and why these initiatives are beneficial to the city system using the following nine case cities as a basis for discussion: Vancouver, New York City, Miami Beach, Sydney, Singapore, Hong Kong, Rio de Janeiro, Copenhagen and Mumbai. It attempts the following four objectives:

• To evaluate the current policy towards urban plastic management across the globe
• To establish the co-benefits of reducing urban plastic in extreme rainfall events
• To evaluate the limitations towards implementing these policies
• To make recommendations for cities experiencing a projected increase in extreme rainfall events

In Section 2, we discuss the legislation regarding plastics and the ocean from the international, national and local levels. In Section 3 we examine the initiatives coastal cities can undertake to combat plastic leakages to the ocean before moving on to the research method in Section 4. In Section 5 we discuss the results found in our case cities. In Section 6, we discuss the co-benefits of implementing these policies before examining the limitations to these policies in Section 7. Finally, in Section 8 we list recommendations for policy makers to minimize plastic leakages to the ocean.

## 2 International agreements and policy

### 2.1 Global agreements

Since the late 20th century there have been international efforts to curb the amount of marine debris in the world's oceans. These agreements underline crucial global issues and efforts and often lead to the creation and enforcement of policy at the national and local levels. In the 2015 G7 summits, the health of the ocean was one of the three key issues of global importance (G7 Germany, 2015). Table 1 lists a selected few international and regional policies from the 1970s-2012. Some of the largest and most impactful agreements have been MARPOL Annex V (shipping waste) and UNCLOS (marine pollution). Both of these conventions are over 30 years old and are limited in their scope. As monitoring is difficult throughout the ocean, in addition to the complicated legalities of international waters, MARPOL is often ignored (Allsopp et al., 2006) and countries create exemptions to the regulations, as the USA has done for their naval vessels (Gold et al., 2013). Furthermore, these conventions are not always practical. MARPOL has denoted special seas where no dumping can occur but regions such as the Caribbean, without appropriate port facilities, can't adhere to these rules (Allsopp et al., 2006).

**Table 1:** A selection of international and regional policy agreements targeting marine debris and plastics. Source: United Nations Environment Programme (UNEP), 2005, Allsopp et al., 2006, Gold et al., 2013, Carroll, 2014.

Date	Policy/Legislation	Purpose
1973/78	International Convention for the Prevention of Pollution from Ships (MARPOL)	Annex V species to prevent the dumping of plastic while at sea and for ports to have adequate facilities to handle waste
1972	London dumping convention	Targets the dumping of land-based waste deliberately at sea
1976	Barcelona convention	Targets the dumping of plastics in the Mediterranean from land and marine sources
1982	UN Convention of the Laws of the Sea (UNCLOS)	Targets the prevention, reduction and control of pollutants in the oceans from land and marine sources
1983	Cartega convention	Targets the dumping of pollutants in the Caribbean from land and marine sources
1992	Helsinki convention	Targets pollution from all land and marine sources
1992	OSPAR	Targets pollution into the North East Atlantic from land and marine sources
1995	FAO code of conduct for responsible fisheries	Targets the management of fishing gear
1995	UNEP global programme of action for the protection of the marine environment from land based activities	Targets pollution from rivers, estuaries and storm drains
2000	EU port reception facilities for ship-generated waste and cargo residues directive	Targets pollution from waste at ports
2005	UN resolution S/60/L.22	Targets the integration of marine debris into national waste management
2008	UN resolution A/60/L.3	Targets the creation of new strategies to tackle lost or abandoned fishing gear
2008	EU marine strategy framework directive	Targets litter in all EU seas based on source and type
2009	UNEP global initiative on marine litter	Creation of twelve regional seas to target marine litter at a regional level. Ties in with the UNEP Regional Seas Programme
2011	Honolulu strategy	Targets the management and monitoring of marine debris. Framework for application
2012	Rio + 20	Targets a reduction in marine debris by 2025
2012	Manila declaration	Targets the reduction of pollution from land-based activities
2012	Global partnership on marine litter	Targets the reduction of land and marine sources as well as reduce impacts on habitats

If signatories of MARPOL Annex V suspect foreign ships of breaking the agreement in their territorial waters, there are

limited legal options for them to pursue, which are costly and lengthy. It is difficult to witness intentional dumping across all territorial waters and as debris moves with the currents, the presence of debris near a vessel is not condemning evidence (Gold et al., 2013). The enforcement of international agreements is not easy. With low fines to dissuade breaking the conventions (Gold et al., 2013) as well as largely ignoring upstream terrestrial sources of debris, the overall effectiveness of the conventions remains questionable.

### 2.2 Regional agreements

There are efforts to reduce marine plastic on regional levels. However, The EU has the only legally binding approach to reducing marine debris at the regional level (Carroll, 2014). As a regulatory entity, the EU can enforce stricter policies for marine debris than a voluntary convention. The EU has also made recent progresses in mandating the removal of microplastics from the cosmetic industries. This will then be transferred into EU nations' national law. In doing so, regional partnerships such as OSPAR (Table 1) in the North East Atlantic is effective as the majority of countries in OSPAR are also in the EU. In the UNEP regional seas programme, some regions perform better than others due to this varying legal monitoring. Outside the EU, regional attempts for managing ocean plastic are limited.

### 2.3 National agreements

There are further management levels for controlling urban plastic spillage into the oceans. Practices can extend from national policies, regional initiatives, municipal ordinances to community action. National governments are responsible for establishing national frameworks for managing the environment as well as setting national quotas and targets. Regional, state or provincial governments set local targets under the national framework. The municipal level acts as a vector for the regional targets and is able to set further local targets. However, there is often tension between these varying levels of governance in managing the environment (Stewart, 1977). The smaller the area of government, the less immediate control they have in creating and enforcing environmental policies due to pressures from higher up governments. This can lead to disagreements in management or a lack of guidance in environmental management. However, the responsibility of management trickles down from the international/regional level to the national and eventually to the smaller local areas tasked with implementing and monitoring progress. These policies include stormwater management, rainfall management, municipal managed recycling, individual/citizen recycling, littering laws, smoking laws, taxes on materials, bans on materials, beach management and marine management. While there are varying levels of policy making, cities and municipal regions remain an important area in implementing policies and directly engaging with the environment.

## 3 Coastal city initiatives

Human settlements concentrate along the world's coast lines. Roughly half of the global population live within 60 km of the coast and nearly three quarters of all large cities are located here (UNEP, 2016). Coastal cities are expected to grow in the future. By 2060, the low elevation coastal zone may grow to 1.4 billion people (Neumann et al., 2015). The population growth, migration to cities and subsequent urban development threatens natural systems such as mangrove swamps and coral reefs, thus jeopardizing the health and resiliency of the coast (Wong et al., 2014). As the number of humans living in coastal areas increases, there needs to be awareness of the pressures their presence puts on the environment.

### 3.1 Systems management

Urban settlements can control the release of plastic during extreme rainfall through systems management. Stormwater systems are put in place to channel rainfall through the city to avoid flooding. However, rainfall in cities often flows directly into adjacent water bodies, either as direct runoff or due to channeling. Stormwater can carry plastic directly into the oceans in coastal cities. Duckett and Repaci (2015) found that plastic frequency on Sydney beaches correlates spatially with the presence of stormwater drains. In addition, the amount of plastic on the beaches is relative to the local area's population size. In the Potomac River, USA Yonkos et al. (2014) found microplastic to be present closest to the urban areas and increased microplastic abundances were found during extreme rainfall events. While this does not suggest stormwater management systems fail to protect the environment from plastic during these events, it does suggest that plastic leaks in higher quantities to the environment during rainfall events. Once in local waterways, cities can manage cleanup systems to remove leaked plastic from the environment. As Gasperi et al. (2014) suggest collecting plastic materials from the waterway not only cleans the urban environment but also allows for planners to examine the total amounts of waste entering the environment.

There is more than one type of stormwater management. However, the most commonplace are traditional combined sewer overflow (CSO) systems, where stormwater is joined with sewage. CSO can lead to the direct flow of this mixed water into the oceans, bringing pollutants with it (Lee and Bang, 2000). Alternatively, there is green infrastructure to handle stormwater including water detention, water retention and water infiltration mechanisms including sustainable urban drainage systems and water sensitive urban design (Taylor, 2013). However, these projects are not globally widespread and it is often costly to upgrade a city's current stormwater system (Stauffer, n.d.). Green infrastructure can account for large scale parkland redevelopment to small scale rainwater capture systems for individual buildings. Green infrastructure can also be implemented to tackle multiple urban problems in addition to flooding such as reducing surface albedos and air quality. Some cities make progress in reducing their traditional CSO systems yet these projects are often time, cost, technically and legislatively intensive (DEC, 2017). Stormwater management is crucial to reducing plastic leakages during extreme rainfall events.

Waste management systems manage the amount of plastic in the urban environment. Landfilling, especially open landfilling near the coast, can allow for plastic leakages into the ocean. However, recycling schemes can control the amount of street plastic. There are both municipal and individual recycling schemes. For example, in Sweden there are municipal recycling schemes in place to collect mixed recyclables including plastic for reprocessing (Sweden.se, 2016). Bottle deposit schemes are individual recycling schemes intending for citizens to return plastic bottles to receive their money back (e.g. New York State, 2016). Both individual and municipal recycling schemes aim to reduce the amount of street litter and plastic that is mismanaged from both the household level and individual level.

### 3.2 Policy management

Outside of systems management, there are policy management techniques to minimize the amount of plastic leakages to the oceans during extreme rainfall events. Economic incentives deter people from intentionally littering. Cities such as Miami Beach and countries such as the UK have littering fines in place to deter street litter (Miami Beach, 2016, BBC, 2016). Fines can be successful in minimizing the amount of litter; the public smoking bans in Japan have reduced the amount of cigarette butts in urban spaces (Ueda et al., 2011). However, even without fines anti-littering campaigns may be enough to dissuade people from littering due to a sense of shame. Grasmick et al. (1991) report a 30% jump in littering guilt in Oklahoma City during the 1980s during anti-littering campaigns. Littering fines in urban areas can dissuade people from leaving plastic accessible to the ocean.

Taxes are instrumental in changing consumer behavior towards plastic. A 15 euro cent tax on plastic bags in Ireland led to a 90% reduction of plastic bag usage in Ireland in the early 2000s (Convery et al., 2007). The tax has successfully removed the widespread use of plastic bags throughout Ireland and has sparked similar taxes globally. Unfortunately, these taxes do not always work efficiently. South Africa has struggled to achieve similar reduction rates in plastic bag usage through taxes (Dikgang et al., 2012). Some cities take it further such as San Francisco banning standard plastic bags forcing the use of alternative bags or biodegradable plastic (Romer, 2010). Taxes alone may not solve the problem of urban plastic but are instrumental in reducing usage.

Education is a key policy area for reducing the amount of plastic easily available to oceans in coastal cities. A plastic consumer will make smarter decisions if they're aware of the consequences of their actions (Sheavly and Register, 2007). Hartley et al. (2015) show that through teaching schoolchildren the problems of marine plastic, the children are more willing to engage with efforts to clean up the oceans. Preparing future citizens is a key to breaking the cycling of poor plastic usage. While people with higher education often report littering less frequently (Eastman et al., 2013) this is not necessarily due to their education regarding marine litter. Furthermore, even if locals are aware of local coastal problems, tourists and visitors contribute to littering (Santos et al., 2005). Education does not need to be limited to marine plastics but rather should focus on living a more sustainable life. However, the education increases the likelihood someone will consider the consequences of their littering but it does not imply that littering will be stopped. Education of marine litter remains a global problem.

## 4 Research method

**Fig. 1:**
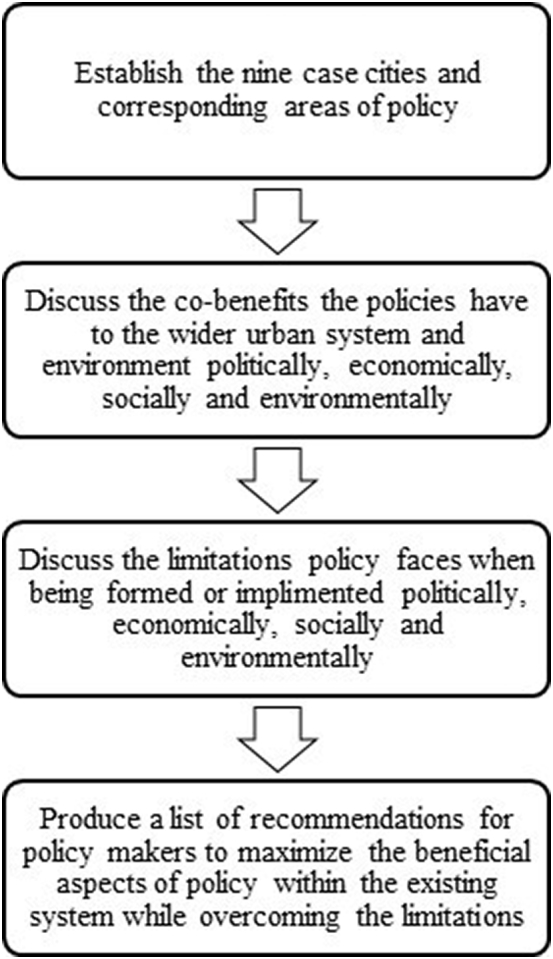
Flowchart of the study’s four major stages of the method.

The research presented here can be separated into four components: data collection and city analysis, the co-benefit of policy, limitations to policy, and recommendations for policy makers. Here, we briefly present the methodology followed for each of these components. The full methodology is summarized in Fig. 1.

### 4.1 Data collection and city analysis

**Fig. 2:**
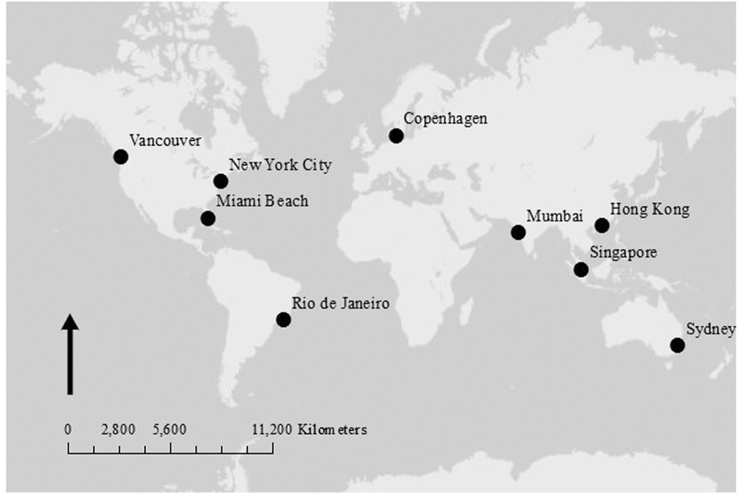
Global map of Vancouver, New York City, Miami Beach, Rio de Janeiro, Copenhagen, Mumbai, Hong Kong, Singapore and Sydney.

To establish a global snapshot of urban coastal policies towards street plastics, this study examined a selection of nine globally spread cities: Vancouver, New York City, Miami Beach, Rio de Janeiro, Copenhagen, Mumbai, Singapore, Hong Kong and Sydney (Fig. 2). These cities represent eight countries with various levels of development. These countries range in the amount of plastic leaked to the marine environment (Jambeck et al., 2015) with China having the highest of 1314.1–3527.9 thousand tonnes annually and Denmark the lowest estimate with 0.3–0.7 thousand tonnes (Table 2). China, India, Brazil and the USA are in the top 20 countries with the highest plastic marine debris entering the ocean. However, while China and India top the list for population, they are near the lowest GDP per capita between the eight countries. These countries represent a range of mismanaged plastic, population and GDP. The cities themselves cover a geographical spread, varying levels of development and legal systems, appearance on the Green Cities Index (Siemens, 2012), and areas of projected increased rainfall (World Bank, 2011, Meehl et al., 2007). The selection of cities was also limited to cities with accessible information on the organization and legal systems of the cities thus two cities were selected in the USA over China, India and Brazil to include more cities in high plastic polluter countries. Projected rainfall changes in these nine cities cover increases in annual rainfall under the CMIP5 and CMIP3 models as well as increases in precipitation intensity projected by the IPCC. This project does not compare rainfall totals between the cities. The rainfall events considered by this project are extreme rainfall events in the top 5% of events.

**Table 2:** Ranking of the eight represented countries.

	Plastic leaked to the marine environment (Thousand T/year) based on Jambeck et al., 2015 (2010 values)	Population (millions) (CIA, 2017)	GDP per capita ($US) (CIA, 2017)
China	1314.1–3527.9	1373.5	14,600
India	89.4–239.2	1266.9	6700
Brazil	70.2–188.6	205.8	14,800
USA	41.0–110.2	324.0	57,300
Australia	2.1–5.6	23.0	48,800
Canada	1.2–3.2	35.4	46,200
Singapore	1.0–2.6	5.8	87,100
Denmark	0.3–0.7	5.6	46,600

Data was extracted from the nine global cities and tabulated into the following sections: stormwater management, rainfall management, municipal managed recycling, individual recycling, littering laws, smoking laws, taxes on materials, bans on materials, beach management and marine management. The policies and laws were extracted from the respective city's governmental websites as well as state/regional and federal government websites. Policies and plans that were proposed but not enacted were not included. Stormwater and rainfall management plans were examined for future plans against rainfall inundation and plastic leakages. Recycling, littering and smoking laws as well as taxes and bans were examined for their policies towards reducing the amount of plastic on the street. Beach and marine management were examined for active policies towards cleaning up the environment.

We recognize that the majority of these policies are not created explicitly to reduce plastic leakages to the ocean. Nonetheless, they are influential in reducing plastic leakages and help tackle marine plastics. The ten categories provide an overall snapshot of urban policies towards the street plastic problem and the policies were discussed in the context of the nine global cities.

### 4.2 The co-benefits of policy

Following the initial discussion of the nine cities and their policies a secondary analysis was performed. The policies are discussed under political, economic, social and environmental co-benefits. Three case examples were used to highlight specific co-benefits; beach tourism for economic benefits, smoking and health for social benefits and CSO for environmental benefits. There was no case example used for political co-benefits due to the lack of politically specific co-benefits of reducing urban street plastic leakages into the ocean. As policy is frequently aimed at tackling multiple problems, the co-benefits discussed are those in which by reducing urban street plastics and leakages there is a direct consequence on the urban environment.

### 4.3 Limitations to policymaking

In the third stage of the analysis, this paper discusses the limitations of policies and policy making in the area of managing urban coastal plastic leakages. Five people were interviewed across four cities: Vancouver, Sydney, New York City and Miami Beach. The interviewees were selected due to their positions in city government, academic work or involvement in non-profit/NGO advocacy. The interviewees' names were altered for the discussion (Table 3). A semi-structure interview style was adopted for its ability to ask standardized question but adapt to each of the interviewees interests, location and professional experience (Longhurst, 2016, Cohen and Crabtree, 2006). The interviews were performed remotely via telephone throughout July 2016. The interviews were partially transcribed for key arguments with all references to the interviewee or interviewee's position title removed. While the interviewees are informed individuals acting within their respective cities, their responsibilities do not encompass every aspect of policy making. Moreover, both interviewees in Miami Beach and New York City are not involved within municipal government. The arguments that all interviews present are therefore subjective in nature to their perceptions and experiences with urban plastics and may not reflect the written reality of the city policies. However, they provide insight from multiple perspectives on the urban plastic problem and the daily reality of their cities.

**Table 3:** Interviewees with altered names and corresponding city location and positions.

Interviewee	City	Position
Vancouver Interviewee A	Vancouver	Metropolitan government
Vancouver Interviewee B	Vancouver	Metropolitan government
Sydney Interviewee	Sydney	Metropolitan government
Miami Beach Interviewee	Miami Beach	Academia
NYC Interviewee	New York City	Non-profit

The interviews identify key limitations towards policy making across the global cities. The discussion is structured similarly into four areas (political, economic, social and environmental), basing its argument on the opinions presented by the interviewees. This study recognizes that the limitations to policymaking vary at different sized jurisdictions. Therefore, this discussion focuses on the limitations to policymaking at the urban municipal scale. It does not focus on limitations faced by state/regional or federal governments.

### 4.4 Recommendations for policy makers

This study presents a list of recommendations for policy makers in formulating future policy towards reducing urban plastic leakages to the marine environment. The recommendations are based on the existing policies, co-benefits and successes of these policies, and the limitations to formulating policy. The list intends to be used a guidance for cities to make future policy alongside additional research for a city's unique location and structuring.

## 5 Results

The following section presents the findings of the city analysis across the nine global cities referring to Table 4. The ten policies were organized into three categories; managing water, managing plastics and managing the environment. Most of the cities had policy in place for each category. There were no clear spatial trends on a hemispherical/regional level or between levels of development. However, the policy in place is place specific and differs between the cities.

**Table 4:** Policy overview of reducing marine plastic in Vancouver, New York City, Miami Beach, Rio de Janeiro, Copenhagen, Mumbai, Hong Kong, Singapore and Sydney.

	Vancouver	New York	Miami Beach	Rio de Janeiro	Copenhagen	Mumbai	Hong Kong	Singapore	Sydney
Stormwater management	Integrated Stormwater Management Plan^17^; Climate Change Adaption Strategy^19^	Sustainable Stormwater Management Plan^5^, ^6^; OneNYC^7^, ^64^	Stormwater Management Master Plan^46^	No master plan but individual waterbodies and planning with the 2016 Olympics^24^, ^29^,^71^	Cloudburst Management Plan^77^; Climate Adaption Plan^76^; runoff management^23^	Government Recovery Plan^67^; Stormwater drainage renovations^49^	Drainage Master Plan Studies^28^	Active, Beautiful Clean water^69^; PUB managing stormwater68,^70^	Decentralised Water Master Plan^8^, ^9^, ^15^
Rainfall management	Integrated Stormwater Management Plan^17^; Climate Change Adaption Strategy^19^	OneNYC^7^, ^64^; Green infrastructure projects^58^, ^66^	Disaster management 44; Stormwater Management Master Plan^46^	No master plan but individual waterbodies and planning with the 2016 Olympics^24^, ^29^,^71^	Cloudburst Management Plan^77^; Climate Adaption Plan^76^	Government Recovery Plan^67^; Disaster Management Monsoon Plan^50^	Drainage Master Plan Studies^28^	Active, Beautiful Clean water^69^; PUB managing stormwater68,^70^	Decetralised Water Master Plan^8^, ^9^, ^15^
Municipal managed recycling	Greenest City Action Plan^20^; Clean Streets Program^18^; recycling guidelines^22^	Formal recycling^37^, ^63^; media campaigns^65^	Formal recycling scheme^47^, ^48^	Formal recycling but informal market larger^85^; 2016 Olympics plans^71^	Waste and Resource Management Plan^78^	No formal recycling	Blueprint for Sustainable Use and formal recycling^31^	Formal recycling schemes^55^, ^57^	Formal recycling scheme^12^
Individual/citizen recycling	Bottle deposit scheme^30^	Bottle deposit scheme^59^	Not to date	Informal market for materials^85^	Bottle deposit scheme^79^	Co-ops and neighborhood initiatives^27^	Producer responsibility towards glass^31^	Not to date	Street recycling^10^, ^13^
Littering laws	Littering fines^16^; Keep Vancouver Spectacular^21^	Littering fines^41^, ^53^	Littering fines^45^	Littering fines^72^	Littering fines^74^	Littering fines^51^	Littering fines^40^; shame campaigns^86^	Littering fines^42^	Littering fines (regional managed)^60^, ^61^
Smoking laws	Bans in indoor public places^38^	Bans in indoor public places and outdoor public parks and beaches^62^	Bans in indoor public places (bars exempt) yet local government can't make further bans^82^	Bans in indoor public places (federal law)^1^	Bans in indoor public spaces^3^	Bans in public places^81^	Bans in indoor public places and outdoor public parks and beaches^36^	Ban in indoor public places and some outdoor public places^56^	Bans in indoor public places and some outdoor public places^11^; free ashtrays^14^
Taxes on materials	Not to date	Plastic bag tax passed the city legislator^39^	Not to date	Not to date	Plastic bag tax^80^	Not to date	Plastic bag tax^33^	Not to date	Not to date
Bans on materials	Not to date	Not to date	Straws on the beach banned^52^	Plastic bags in stores banned^25^	Not explicit	Plastic bags less than 50 μm banned^84^	Not explicit	Not to date	Not to date
Beach management	Campaigns organized outside of city government. Example: Great Canadian Shoreline Cleanup^83^	Campaigns organized outside of city government. Example: American Littoral Society^2^	Adopt-a-beach^43^; partnerships with ECOMB^32^	Cleanup projects through the city^4^, ^71^	Not Explicit	Not explicit	Cleanup projects through the city^34^	Localized campaigns through the city^54^, ^75^	Coastal cleanup^73^
Marine management	Not explicit	Education programs and cleanups^65^	Not explicit	Cleanup projects through the city^4^, ^71^	Harbor cleanup with reducing CSO^23^, ^26^	Not explicit	Cleanup projects^34^; follow international laws^35^	Not explicit	Active harbor cleaning^73^

1 Alves, L. (2014), The Rio Times, ‘Ample anti-smoking law implemented in Brazil’ (WWW), Available at: http://riotimesonline.com/brazil-news/rio-politics/ample-anti-smoking-law-implemented-in-brazil/; Accessed 2/8/16.

2 American Littoral Society (n.d.) ‘Marine debris’ (WWW), Available at: http://www.littoralsociety.org/index.php/programs/habitat-restoration/marine-debris; Accessed 2/8/16.

3 Americans for Nonsmokers Rights (2014) ‘Denmark’ (WWW), Available at: http://www.no-smoke.org/goingsmokefree.php?id=635; Accessed 2/8/16.

4 Byrnes, M. (2014), City Lab, ‘Rio's relentless beach pollution’ (WWW), Available at: http://www.citylab.com/politics/2014/03/rios-relentless-beach-pollution/8729/; Accessed 2/8/16.

5 City of New York (2008) *Sustainable Stormwater Management Plan*, New York City: PlaNYC.

6 City of New York (2012) Sustainable Stormwater Management Plan: Progress Report October 2012, New York City: PlaNYC.

7 City of New York (2016) ‘OneNYC’ (WWW), Available at: http://www1.nyc.gov/html/onenyc/index.html; Accessed 2/8/16.

8 City of Sydney (2012a) *Decentralised Water Master Plan 2012–2030*, Sydney: City of Sydney.

9 City of Sydney (2012b) *Decentralised Water Master Plan: WSUD and Stormwater Infrastructure Report*, Sydney: City of Sydney.

10 City of Sydney (2014) ‘Cash for containers’ (WWW), Available at: http://www.cityofsydney.nsw.gov.au/live/waste-and-recycling/clean-streets/envirobank-reverse-vending-machines/cash-for-containers; Accessed 2/8/16.

11 City of Sydney (2016a) ‘Cigarette butts’ (WWW), Available at: http://www.cityofsydney.nsw.gov.au/live/waste-and-recycling/clean-streets/yuk-cigarette-butts; Accessed 2/8/16.

12 City of Sydney (2016b) ‘Recycling’ (WWW), Available at: http://www.cityofsydney.nsw.gov.au/live/waste-and-recycling/recycling; Accessed 2/8/16.

13 City of Sydney (2016c) ‘Recycling machines’ (WWW), Available at: http://www.cityofsydney.nsw.gov.au/live/waste-and-recycling/clean-streets/envirobank-reverse-vending-machines; Accessed 2/8/16.

14 City of Sydney (2016d) ‘Smoking in public places’ (WWW), Available at: http://www.cityofsydney.nsw.gov.au/community/health-and-safety/smoking-in-public-places; Accessed 2/8/16.

15 City of Sydney (n.d.) *Sustainable Sydney 2030: Snapshot*, Sydney: City of Sydney.

16 City of Vancouver (2016a) ‘Abandoned garbage and illegal dumping’ (WWW), Available at: http://vancouver.ca/home-property-development/abandoned-garbage-and-illegal-dumping.aspx; Accessed 2/8/16.

17 City of Vancouver (2016b) ‘Citywide integrated rainwater management plan’ (WWW), Available at: http://vancouver.ca/home-property-development/city-wide-integrated-stormwater-management-plan.aspx; Accessed 2/8/16.

18 City of Vancouver (2016c) ‘Clean Streets program’ (WWW), Available at: http://vancouver.ca/people-programs/clean-streets-program.aspx; Accessed 2/8/16.

19 City of Vancouver (2016d) ‘Climate change adaption strategy’ (WWW), Available at: http://vancouver.ca/green-vancouver/climate-change-adaptation-strategy.aspx; Accessed 2/8/16.

20 City of Vancouver (2016e) ‘Greenest city action plan’ (WWW), Available at: http://vancouver.ca/green-vancouver/greenest-city-action-plan.aspx; Accessed 2/8/16.

21 City of Vancouver (2016f) ‘Keep Vancouver spectacular’ (WWW), Available at: http://vancouver.ca/people-programs/keep-vancouver-spectacular.aspx; Accessed 2/8/16.

22 City of Vancouver (2016g) ‘Recycling guidelines and service’ (WWW), Available at: http://vancouver.ca/home-property-development/recycling.aspx; Accessed 2/8/16.

23 Clauson-Kaas, J., Sorensen, S., Johansen, N.B. and Nielsen, J.B. (2008) ‘Run-off management in Copenhagen Harbour’, *11th International Conference on Urban Drainage*, Edinburgh, Scotland, 2008.

24 Columbia University (n.d.) ‘Water management system of the Rio de Janeiro city’ (WWW), Available at: http://engineering.columbia.edu/files/engineering/design-water-resource06.pdf; Accessed 2/8/16.

25 De Biagio, F. (2010), 20BN America, ‘Plastic bag restrictions come into force in Rio de Janeiro’ (WWW), Available at: http://www.bnamericas.com/en/news/petrochemicals/Plastic_bag_restrictions_come_into_force_in_Rio_de_Janeiro; Accessed 2/8/16.

26 Denmark.dk (2016) ‘Swimming in Copenhagen Harbor’ (WWW), Available at: http://denmark.dk/en/green-living/copenhagen/swimming-in-copenhagen-harbour; Accessed 2/8/16.

27 Dharavi Market (2012) ‘Recycling Mumbai’ (WWW), Available at: http://www.dharavimarket.com/community/Recycling/; Accessed 2/8/16.

28 Drainage Services Department (2014) ‘Drainage Master Plan Studies and Drainage Studies’ (WWW), Available at: http://www.dsd.gov.hk/EN/Flood_Prevention/Long_Term_Improvement_Measures/Drainage_Master_Plan_Studies_and_Drainage_Studies/index.html; Accessed 2/8/16.

29 Duarte, M.A., da Fonseca, P.L. and Pimentel da Silva, L. (2011) ‘Sustainable systems management- Opportunities and challenges for the implementation of the Urban Master Plan regarding stormwater management from the City of Rio de Janeiro, Brazil’, *12th International Conference on Urban Drainage*, Porto Alegre, Brazil, 11–16 September 2011.

30 Encorp (2016) ‘Deposits, fees, and other container types’ (WWW), Available at: https://www.return-it.ca/beverage/products/; Accessed 2/8/16.

31 Environment Bureau (2013) Hong Kong Blueprint for Sustainable Use of Resources 2013–2022, Hong Kong; Environment Bureau.

32 Environmental Coalition of Miami and the Beaches (ecomb) (2010) ‘Litter prevention’ (WWW), Available at: http://ecomb.org/programs/litter-prevention/; Accessed 2/8/16.

33 Environmental Protection Department (2015) ‘Environmental levy scheme on plastic shopping bags’ (WWW), Available at: http://www.epd.gov.hk/epd/english/environmentinhk/waste/pro_responsibility/env_levy.html; Accessed 2/8/16.

34 Environmental Protection Department (2016) ‘Clean Shorlines’ (WWW), Available at: http://www.epd.gov.hk/epd/clean_shorelines/index-2.html; Accessed 2/8/16.

35 GovHK (2015) ‘Environmental laws and regulations’ (WWW), Available at: http://www.gov.hk/en/residents/environment/business/laws.htm; Accessed 2/8/16.

36 GovHK (2016) ‘Tobacco control’ (WWW), Available at: http://www.gov.hk/en/residents/health/addictions/smoking/tobaccocontrol.htm; Accessed 2/8/16.

37 Grow NYC (2015) ‘What to recycle in New York City’ (WWW), Available at: http://www.grownyc.org/recycling/whattorecycle; Accessed 2/8/16.

38 Health By-Law No. 9535 (2015), Vancouver: Council of the City of Vancouver.

39 Heins, S. (2016) ‘NYC plastic bag fee postponed until next year’ (WWW), Available at: http://gothamist.com/2016/06/22/plastic_bag_valentines.php, Accessed 2/8/16.

40 Hong Kong Police Force (2016) ‘Laws of Hong Kong to be observed’ (WWW), Available at: http://www.police.gov.hk/ppp_en/11_useful_info/filming/laws.html; Accessed 2/8/16.

41 Justia (2006) ‘2006 New York Code- Littering Prohibited’ (WWW), Available at: http://law.justia.com/codes/new-york/2006/new-york-city-administrative-code-new/adc016-118_16-118.html; Accessed 2/8/16.

42 Khew, C. (2015), The Straits Times, ‘Current measures against littering in Singapore’ (WWW), Available at: http://www.straitstimes.com/singapore/environment/current-measures-against-littering-in-singapore; Accessed 2/8/16.

43 Miami Beach (2016a) ‘Adopt-a-Beach’ (WWW), Available at: http://www.miamibeachfl.gov/green/default.aspx?id=63494; Accessed 2/8/16.

44 Miami Beach (2016b) ‘Flood safety awareness’ (WWW), Available at: http://miamibeachfl.gov/dEM/scroll.aspx?id=59285; Accessed 2/8/16.

45 Miami Beach (2016c) ‘Keep Miami Beach clean’ (WWW), Available at: http://www.miamibeachfl.gov/litter/; Accessed 2/8/16.

46 Miami Beach (2016d) ‘Public works- storm water’ (WWW), Available at: http://miamibeachfl.gov/publicworks/scroll.aspx?id=27280; Accessed 2/8/16.

47 Miami Beach (2016e) ‘Recycling’ (WWW), Available at: http://www.miamibeachfl.gov/recycle/; Accessed 2/8/16.

48 Miami Beach (2016f) ‘Recycling ordinance’ (WWW), Available at: http://www.miamibeachfl.gov/recycle/scroll.aspx?id=71525; Accessed 2/8/16.

49 Ministry of Environment, Forest and Climate Change (n.d.) ‘Storm water drainage’ (WWW), Available at: http://forestsclearance.nic.in/writereaddata/Addinfo/0_0_1112123912121BRIMSTOWADreportonStormWaterDrainage.pdf; Accessed 2/8/16.

50 Municipal Corporation of Greater Mumbai (2016a) ‘Disaster Management’ (WWW), Available at: http://www.mcgm.gov.in/irj/portal/anonymous/qldma?guest_user=english#; Accessed 2/8/16.

51 Municipal Corporation of Greater Mumbai (2016b) ‘Greater Mumbai Cleanliness and Sanitation Byelaws 2006’ (WWW), Available at: http://www.mcgm.gov.in/irj/portal/anonymous/qlblaw; Accessed 2/8/16.

52 Munzenrieder, K. (2012), Miami New Times, ‘Miami Beach has banned plastic straws’ (WWW), Available at: http://www.miaminewtimes.com/news/miami-beach-has-banned-plastic-straws-6547654; Accessed 2/8/16.

53 National Conference of State Legislateres (NCSL) (2014) ‘States with littering penalties’ (WWW), Available at: http://www.ncsl.org/research/environment-and-natural-resources/states-with-littering-penalties.aspx; Accessed 2/8/16.

54 National Environment Agency (2015) ‘Clean Singapore Learning Trail (Beaches)’ (WWW), Available at: http://www.nea.gov.sg/events-programmes/programmes/schools-youth/clean-singapore-learning-trail-beaches; Accessed 2/8/16.

55 National Environment Agency (2016a) ‘National Recycling Programme’ (WWW), Available at: http://www.nea.gov.sg/energy-waste/3rs/national-recycling-programme; Accessed 2/8/16.

56 National Environment Agency (2016b) ‘Smoking prohibited’ (WWW), Available at: http://www.nea.gov.sg/public-health/smoking; Accessed 2/8/16.

57 National Environment Agency (2016c) ‘Waste minimization and recycling’ (WWW), Available at: http://www.nea.gov.sg/energy-waste/3rs; Accessed 2/8/16.

58 National Resource Defense Council (n.d.) ‘New York, New York: A case study of how green infrastructure is helping manage urban stormwater challenges’ (WWW), Available at: https://www.nrdc.org/sites/default/files/RooftopstoRivers_NewYork.pdf; Accessed 2/8/16.

59 New York State Department of Environmental Conservation (2016) ‘Frequently asked questions about the bottle bill’ (WWW), Available at: http://www.dec.ny.gov/chemical/57687.html; Accessed 2/8/16.

60 NSW EPA (2015) ‘Litter laws’ (WWW), Available at: http://www.epa.nsw.gov.au/litter/laws.htm; Accessed 2/8/16.

61 NSW EPA (2016) ‘Litter fines’ (WWW), Available at: http://www.epa.nsw.gov.au/litter/fines.htm; Accessed 2/8/16.

62 NYC Coalition for a Smoke-Free City (n.d.) ‘Existing legislation’ (WWW), Available at: http://nycsmokefree.org/existing-legislation; Accessed 2/8/16.

63 NYC Department of Sanitation (2015) ‘Zero waste’ (WWW), Available at: http://www1.nyc.gov/assets/dsny/zerowaste/residents.shtml; Accessed 2/8/16.

64 NYC Environmental Protection (2015a) One New York City: One Water, New York City: NYC Environmental Protection.

65 NYC Environmental Protection (2015b) Trash Free NYC Waters: A Plan to Reduce Marine Debris through a Media Campaign, New York City: NYC Environmental Protection.

66 NYC Environmental Protection (2016) ‘Green Infrastructure Plan and reports’ (WWW), Available at: http://www.nyc.gov/html/dep/html/stormwater/nyc_green_infrastructure_plan.shtml; Accessed 2/8/16.

67 Phatak, J. (n.d.) ‘Management of urban floods in Mumbai, India’ (Powerpoint Presentation), Unknown location and date, provided by UNISDR, May 2012.

68 PUB (2014) Managing Stormwater for Our Future, Singapore: PUB.

69 PUB (2016a) ‘Active, Beautiful, Clean Waters Programme’ (WWW), Available at: https://www.pub.gov.sg/abcwaters; Accessed 2/8/16.

70 PUB (2016b) ‘Stormwater management’ (WWW), Available at: https://www.pub.gov.sg/drainage/stormwatermanagement; Accessed 2/8/16.

71 Rio 2016 (2013) Sustainability Management Plan: Rio 2016 Olympic and Paralympic Games, Rio de Janeiro: Rio 2016.

72 Rio Uncovered (2013) ‘Fines for littering in Rio de Janeiro come into force’ (WWW), Available at: http://riouncovered.com/fines-for-littering-in-rio-de-janeiro-come-into-force/; Accessed 2/8/16.

73 Roads and Maritime (2014) ‘Sydney Harbor environmental services’ (WWW), Available at: http://www.rms.nsw.gov.au/about/environment/sustainability/sydney-harbour.html; Accessed 2/8/16.

74 Rychla, L. (2016), Copenhagen Post, ‘Danes want stiffer fines for littering’ (WWW), Available at: http://cphpost.dk/news/danes-want-stiffer-fines-for-littering.html; Accessed 2/8/16.

75 Tay, E. (2014), Green Future Solutions, ‘Insights on marine trash in Singapore’ (WWW), Available at: http://www.greenfuture.sg/2014/08/12/insights-on-marine-trash-in-singapore/; Accessed 2/8/16.

76 The City of Copenhagen (2011) Copenhagen Climate Adaption Plan, Copenhagen: City of Copenhagen.

77 The City of Copenhagen (2012) Cloudburst Management Plan 2012, Copenhagen: City of Copenhagen.

78 The City of Copenhagen (2014) Resource and Waste Management Plan 2018, Copenhagen: City of Copenhagen.

79 The City of Copenhagen (2016) ‘How do I recycle bottles?’ (WWW), Available at: http://international.kk.dk/artikel/how-do-i-recycle-bottles; Accessed 2/8/16.

80 The Danish Ecological Council (n.d.) Fact Sheet: Tax on Plastic Bags, Copenhagen: The Danish Ecological Council.

81 Tobacco Control Laws (2015) ‘Country details for India’ (WWW), Available at: http://www.tobaccocontrollaws.org/legislation/country/india/summary; Accessed 2/8/16.

82 US Legal (2016) ‘Smoking regulations in Florida’ (WWW), Available at: http://smoking.uslegal.com/smoking-regulations-in-florida/; Accessed 2/8/16.

83 Vancouver Aquarium (2016) ‘Great Canadian shoreline cleanup’ (WWW), Available at: http://shorelinecleanup.ca/; Accessed 2/8/16.

84 Vyas, S. (2010), The Times of India, ‘Plastic bags: No ban, only penalty’ (WWW), Available at: http://timesofindia.indiatimes.com/city/mumbai/Plastic-bags-No-ban-only-penalty/articleshow/5414807.cms; Accessed 2/8/16.

85 Wilkes, C. (2015), Rio on Watch, ‘Recycling in Rio de Janeiro: An overview’ (WWW), Available at: http://www.rioonwatch.org/?p=21419; Accessed 2/8/16.

86 Worland, J. (2015), Time, ‘Hong Kong anti-littering campaign uses DNA from trash to shame people’ (WWW), Available at: http://time.com/3890499/hong-kong-littering-campaign/; Accessed 2/8/16.

### 5.1 Managing water

For coastal cities, managing their water is integral to the functioning of the city. All of the nine cities have a plan in place for stormwater management and rainfall management (Table 4). Rio de Janeiro was the only city to have water management as part of a goal for development towards an event, the 2016 Olympic Games. The other cities have developed their plans to respond to the problems of rainfall inundation and overall sustainability in their cities. While most plans are comprehensive of the entire city, Hong Kong's stormwater and rainfall management is structured for the city and specific city regions. Some drainage master plans are for specific regions in the city allowing for a more detailed study to be done throughout Hong Kong. While all the cities have a plan to manage stormwater and rainfall, the plans are not uniform in their scope.

Although the plans for stormwater and rainfall management are important for the future of the cities, the legal framework and structuring of urban governance weakens the policies. New York City's Sustainable Stormwater Management Plan is no longer being actively implemented but instead is being reincorporated under OneNYC due to the legislative changes made at the state and local level (Table 4). These policies are subject to change with new administrations.

The water management plans are often well placed within larger policy contexts. For example, Singapore's Active, Beautiful, Clean Water program incorporates cleaning waterways not only for reduced pollution but also for community access to greenspace and city beautification. Furthermore, Vancouver, New York City, Rio de Janeiro, Copenhagen and Sydney explicitly link their water management with climate change or general sustainable management. This places good stormwater and rainfall management in the discourse of a sustainable, healthy city for the future, encouraging further developments.

A few of the policies target niche components of rainfall and stormwater management; extreme downpours and flooding. Copenhagen's Cloudburst Management Plan (Table 4) “outlines the methods, priorities, and measures recommended for the area of climate adaption.

including extreme rainfall. With this plan, “[the city has] taken decisive steps forward to protect Copenhagen against high-intensity rain like the ones witnessed in August 2010 and again in July and August 2011” (p.5). The plan recognizes that despite future financial investments in expanding traditional sewer systems, the cost from rainfall inundation would be too high (European Climate Adaption Platform, 2016). Instead, the plan utilizes both altering the sewer network and focusing on surface water retention. It also expands responsibility for the city to manage the public space, private landowners to manage private property and the water company to manage the public piping (Therkildsen, 2015). This plan actively engages the entire city in making Copenhagen more resilient towards extreme rainfall events and 100-year level floods.

Both Mumbai and Miami Beach have specific plans for disaster management with rainfall and flooding, respectively (Table 4). While both of these cities focus on the human and capital losses during these disasters, they nonetheless recognize the environmental consequences for the surrounding waterways. These policies are not exclusive to rainfall however, both recognize the role storm surges play in potential problems with storms. With the future hazard of increased rainfall and intensity, these cities, if not being more resilient to rainfall, allow for increased flood and rainfall specific policy to be expanded upon. This also highlights this city government's recognition of rainfall and flooding as a specific problem.

There are two ways in which the nine cities are moving towards stormwater and rainfall management: reducing CSO and expanding on green infrastructure. CSO's historically were able to handle the influx of precipitation in cities. However, with the increasing volumes and intensity of rainfall, CSOs struggle to effectively handle storms. For several years, Copenhagen has redeveloped their CSO system to minimize the amount of pollution that enters its coastal waters (Table 4^22^). Sydney has also made large efforts to remove CSO systems from the city. So, while these systems are still present, their influence in handling stormwater has decreased with new water management techniques. Vancouver is working towards British Columbia's goal of eliminating CSO by 2050 (City of Vancouver, 2016b). Furthermore, New York City has seen an increase in CSO capture rates from 30% in the 1980s to over 80% today (NYC Environmental Protection, 2016a). Replacing CSO with separate systems or diverting water flow away from CSOs is the main hard infrastructure these cities do to handle stormwater.

Green infrastructure provides a flexible future for these cities to reduce the effects of rainfall on their systems. Green infrastructure attempts to re-naturalize the landscape and reduce the amount of runoff during rainfall events, and thus the amount of street litter carried by rain. Green infrastructure can be implemented on a large scale for example a park or smaller scales such as individual buildings. New York City was the only city to have a specific green infrastructure plan (Table 4) yet most cities recognize the importance of rebuilding a naturalized landscape. New York City's green infrastructure is targeted towards stormwater pollution and measures its success not just on reductions of flow to the CSO but also the quality of the effluent. Green roofs are well placed to help tropical cities like Hong Kong to reduce the sudden inundation from extreme rainfall events (Hui and Chu, 2009). Vancouver utilizes a wide array of green infrastructure such as rain gardens as a way to filter stormwater before it enters the drains (City of Vancouver, 2016a). Green infrastructure is integral to Copenhagen's rainfall strategies. Designing public spaces to store stormwater, implementing pervious pavements, and constructing rain gardens to filter stormwater are some of the many green infrastructure programs the city has embraced to handle extreme rainfall (Haghighatafshar et al., 2014). Green infrastructure can help ameliorate the load of stormwater when traditional systems are overwhelmed.

### 5.2 Managing plastic

The cities have a mixed response when it comes to managing the amount of plastic in the city. Practical initiatives such as bans, taxes and deposits are not shared by all the cities. All cities but Mumbai manage a formal recycling system. Of the remaining eight cities, all but Miami Beach place recycling plans and strategies explicitly in the context of future sustainability. Again, the 2016 Olympics pressured Rio de Janeiro to re-strategize their waste management and create future recycling plans as opposed to diverting most waste to landfill (Table 4). Sustainability is at the forefront of the city policies when it comes to formally managing waste and plastics. Both Vancouver and New York City take their plans further by setting targets for waste management; New York City aiming for zero waste to landfill by 2030 and Vancouver aiming for a 50% reduction in waste to landfill or incineration from 2008 values by 2020. These goals, while ambitious and nearly 10 years old (starting in 2007 and 2009 respectively) set a clear focus for recycling and waste management. Vancouver's strategy is also linked to the province-wide waste strategy for British Columbia (City of Vancouver, 2016c). Vancouver, in addition, furthers its commitment to waste management by employing those in need of work to maintain clean streets and remove street litter through the Clean Streets Program. While almost all of the cities have a formal process of handling plastics, some cities have more defined policies and targets for reducing the amount of unsustainably managed waste.

There isn't a consistent strategy for incentivizing individual citizen recycling in the cities. Bottle deposits are present in three cities; Vancouver, New York City and Copenhagen (Table 4). Both Vancouver's and New York City's deposits emerged from their province/state government and Copenhagen's deposit is a national law. Vancouver covers most drinking containers while Copenhagen only has deposits on some glass bottles, cans and plastic bottles and New York City differentiates deposits by product. British Columbia diverted 1.5 billion containers from landfill in 2010 (Bottle Bill Resource Guide, 2014) and New York State has seen a decrease in 70% of roadside container litter since its development in the 1980s (New York State Department of Environmental Conservation, 2016). However, bottle laws are not the only form of individual recycling in the cities. The two developing cities, Rio de Janeiro and Mumbai, rely on the informal market for recycling as individuals can resell valuable products for an income. However, cheap plastics are often ignored in this market and Rio de Janeiro (Table 4) recognizes that the development of the formal recycling system will divert income away from this market. In addition, the plastic recycling companies in Rio de Janeiro only had a 16% capacity for all the plastic waste (Pacheco et al., 2012). Hong Kong focuses on glass bottles leaving the responsibility towards the producers to collect and reprocess containers. Singapore and Miami Beach were the two cities that had no economic individual incentive towards recycling.

Every city has laws against littering in the city punishable with fines. The fines vary in cost and severity. Due to differences in currency and median incomes it is difficult to determine an order of the height of fines per city. However, both Singapore and Miami Beach appear to take a particularly harsh approach to littering. Miami Beach's first offense is a fine of US$1500 going up to US$3500 with continued littering while Singapore's initial offense carries a maximum fine of US$1485 (SG$2000) going up to US$7426 (SG$10,000) on subsequent fines. Singapore can also issue Corrective Work Orders to litterers forcing them to clean public areas. In 2014, 688 of these orders were issued (Table 4). Furthermore, Hong Kong has taken a similar shame campaign using the DNA found on items of litter to generate a supposed sketch of the litterer to be portrayed around the city. On a softer side, Vancouver sponsors a community campaign, Keep Vancouver Spectacular to encourage local communities to monitor and clean their areas. However, none of the cities have a specific police litter task force to monitor these laws. Therefore, it is difficult to measure the effectiveness of these laws in deterring littering. Rather each city's police force place different priorities on upholding the anti-littering laws with Singapore being the most observant and Mumbai and Rio de Janeiro the least.

Every city has a ban on smoking (Table 4). All the laws cover indoor public places, mostly to cut the consequences of second hand smoke to the cities' populations. However, New York City, Hong Kong, Singapore and Sydney include bans on outdoor public places, including neighborhood areas, parks and beaches. Not only are these ordinances intended to further reduce the effects of second hand smoke but also to reduce the amount of cigarette butts found in the local environment. The laws towards smoking and cigarettes is still mostly focused on human health but is moving towards environmental concerns.

The most practical measures towards reducing street plastics, bans and taxes, are the most inconsistent across the cities. These ordinances would target the top of the waste pyramid, prevention. Only two cities have a tax on plastics, specifically plastic bags; Copenhagen and Hong Kong. The remaining seven cities have no explicit consumer tax on using plastics. Unlike deposit schemes, the tax on plastic bags is non-refundable and is a direct added cost to the consumer.

### 5.3 Managing the environment

Beach management and marine management are active ways in which the city can spread education and clean up the environment. Of the nine cities, only Mumbai was not explicit in the government's role in these management areas. Three cities; Rio de Janeiro, Hong Kong and Sydney were explicitly involved in both marine and beach management.

Beach cleanup campaigns appeared differently across the cities. Rio de Janeiro, again influenced by the 2016 Olympics, has localized cleanup projects sponsored by the government in the beaches and coastal margins of the city. Similarly, Singapore has localized cleanup campaigns, including those aimed at children, to maintain beaches as well as standardized government cleanup of beaches. However, Singapore does not denote a single agency to be in charge of managing the amount of plastic that ends up in the marine environment and the beaches. In Sydney, the NSW government actively cleans the harbor on behalf of the city. Hong Kong and Miami Beach have the most comprehensive beach cleanup campaigns. Hong Kong has a Clean Shorelines initiative where a working group sponsored by the government attempts to study the marine plastics, identify their sources and identify potential cleanup methods while helping coordinate community action on cleaning up the beaches. In contrast, Miami Beach has partnered with the Environmental Coalition of Miami and the Beaches, a county wide organization that attempts to protect all the beaches in the Miami area. Through this community sponsored adopt-a-beach programmes as well as cleanup events and education campaigns are utilized in Miami Beach as well as standardized for the surrounding area. Of the existing beach management policies, the cities differ on the extent and scope of their involvement.

While the city government may not be active in beach management campaigns, NGOs, non-profits and community action often fills the gap left by the city government. In addition, other governments may be charged in handling beach cleanup campaigns. In Miami Beach, the state of Florida has statewide cleanups to increase awareness of the marine pollution problems (Florida Department of Environmental Protection, 2012, University of Florida, 2006). In Vancouver and New York City, Great Canadian Shoreline Cleanup and American Littoral Society are just two of many organizations targeted towards the cleaning, education and advocating about marine plastics and marine debris. While a city may not be active in beach management there may be organizations present engaging in the campaigns.

While marine management is difficult to define specifically for coastal plastics, several policies were identified across the cities. Controlling sewage is important in several cities. Rio de Janeiro is attempting projects to reduce sewage leakage to the Guanabara Bay through infrastructure developments. Copenhagen has closed 55 of the 93 CSO leading to the harbor so that today Copenhagen harbor has some of the cleanest water in the European Union. Hong Kong actively advertises their compliance with international protocols including marine pollution. In addition, they have strict ordinances on the condition of sewage effluents in the surrounding waters. However, there is active cleaning for debris in some of the cities. In Sydney harbor, beyond managing the beaches and CSO outlets, the NSW government also removes floating debris from the harbor; roughly 3500 m^3^ of rubbish is collected from the water and beaches (Table 4^73^). In New York City, just over 1000 m^3^ of debris was collected in 2012 (Table 4^65^). Furthermore, there may be individual cleanup projects for specific waterways within a city. In New York City, there is a specific plan in place to prevent debris in the Newtown Creek (NYC Environmental Protection, 2016b). Some cities were more explicit in how they manage their marine environment. However, in those that do state their management, focus is placed on sewage controls and removing floating debris.

## 6 The co-benefits of policy

The following discussion of the co-benefits of policy is organized under the following headings: Political, Economic, Social and Environmental. The policies cities adopt to prevent plastic leakages often create multiple benefits around the city. The strength of these policies is therefore not in its ability to solely prevent plastic in the environment, but to improve the entire city system.

### 6.1 Beach economics

By reducing plastics in the environment, cities can experience long term economic benefits. Litter cleanup and litter prevention campaigns are costly to cities (Monroe, 2013, Schneider et al., 2011). The lack of green infrastructure in most cities leads to increased rainfall inundation. This flooding disproportionately affects the poor living in poor drainage areas (Braun and Abheuer, 2011, Jha et al., 2012) as well as damaging city infrastructure (Hallegatte et al., 2013). As tourism grows, however, beach economies are particularly beneficial to city economies.

Clean beaches, parks and open space have a large economic value for cities and states. Coastal tourism in the United Kingdom is estimated around US$8–12.5 billion a year (Mouat et al., 2010). The regional value of Australian beaches near the Great Barrier Reef comes to roughly US$447.3 million (Rolfe and Gregg, 2012). In Australia, Canada, New Zealand and the USA 24% of the marine economy is based in tourism worth around US$207.33 billion in 2008 (McIIgorm et al., 2011). The beach economy is a vital resource for coastal regions. However, determining the exact value of the economy is difficult. Creating a value for beaches requires including or excluding tourism spending, business revenue, housing premiums from beach access, the aesthetic and cultural value of beaches, the value of beach ecosystem services, etc. Each estimate may use a different selection of criteria to value the beach economy. Despite the difficulty in valuing a beach, it is undeniable that beaches have the potential to bring money to an area through tourism.

Plastic and marine debris have a direct negative impact on the economic viability of beaches. For example, debris on Swedish beaches can cost the surrounding areas between 1 and 5% of tourism, a worst-case scenario of roughly US$19.7 million a year (UNEP, 2009). In 1988, marine litter wash-ups cost the state of New Jersey between US$379 million-US$3.6 billion in lost tourism revenue and the state of New York lost US$950 million-US$2 billion (Committee on the Effectiveness of International and National Measures to Prevent and Reduce Marine Debris and Its Impacts, 2008). Tourists avoid beaches that have litter, although it is difficult to estimate at what point litter deters tourists (Mouat et al., 2010). Therefore, the size of the beach industry weights the importance cities place on actively removing plastic from the marine environment.

By reducing urban plastics, cities will be able to reduce costs associated with cleaning beaches and increase the beach revenue itself. Leggett et al. (2014) examined the benefits of reducing debris in six beaches near the mouth of the Los Angeles River. By reducing debris 75%, visitation to the beaches is estimated to increase 43% thus bringing in a revenue of US$53 million in benefits to the surrounding communities. Furthermore, reducing debris by 25% could see the residents of Orange County, California benefit US$32 million a year over the three peak summer months. In cities reliant on tourism, the increased revenue from cleaner beaches can be reinvested in further sustainability measures such as flood defenses against rising sea levels. By reducing visible debris on beaches, cities can experience great financial benefits.

### 6.2 Social quality of life and smoking

Reducing plastics in city waterways can foster a better social atmosphere. Litter cleanup creates unique employment opportunities for cities (Washington State Department of Ecology, n.d, Union County Government North Carolina, n.d). Greenspaces and green infrastructure may also foster a healthier environment. While the direct causality of greenspaces and wellbeing are not conclusive (Lee and Maheswaran, 2011), greenspaces may encourage increased physical activities (Coombes et al., 2010, Nutsford et al., 2013, Thompson et al., 2016) as well as reduced stress levels (Nutsford et al., 2013, Thompson et al., 2016). Reducing plastics presents an opportunity to create a cleaner city. Where cities use plants in their green infrastructure is also encourages a greener city.

By reducing smoking in public places, the presence of cigarette butts is removed from the environment while citizens may be discouraged from smoking and improve their health. As teenagers and young adults develop, the stricter the controls on smoking in public places and the smaller visual presence smoking has in a community, the less likely it is teenagers will smoke (Wakefield et al., 2000, Alesci et al., 2003). As less citizens take up smoking, there will be less cigarette butts produced. In addition, there will be less health associated risks with smoking and second hand smoke further reducing hospital burdens and costs. Smoking bans can have high acceptance rates and lead to reduced smoking rates. In Ireland, 46% of Irish smokers polled agree the public ban on smoking would encourage them to quit while 80% of quitters polled agree the law helped them quit and 88% polled agree the law helped them stay clean (Fong et al., 2006). With smoking reduced in public places, there are less cigarette butts on the street with potential to enter local waterways and there are lower rates of smoking among future generations and less smoking related health costs. However, laws alone are not always enough to curb smoking. People continue to smoke in public places regardless of fines and strict enforcement in the immediate of a ban is necessary to reshape public habits (Yang et al., 2016). In the long run, anti-smoking laws can dramatically improve the social lives of cities through cleaner streets and less smoking and second hand smoke related health issues.

### 6.3 Environmental benefits and CSOs

Policies that reduce urban plastics can ultimately support a clean urban environment. Urban greenspaces have a variety of benefits from increasing housing values (Bolund and Hunhammar, 1999, Sadeghian and Vardanyan, 2013), reducing the urban heat island effect (Solecki et al., 2005), and supporting local flora and fauna (Sadeghian and Vardanyan, 2013). Reducing plastics also presents cities with an opportunity to increase recycling and reduce their need for landfilling. The environment benefits when plastics are reduced from cities' waterways.

CSOs are undoubtedly harmful to urban environments. Through sewage overflow, the water surrounding cities can receive pathogens, pharmaceutical chemicals, nitrogen and phosphorous, human waste and wrongfully disposed sanitary products (Veronesi et al., 2014) in addition to the street litter and plastics entering with stormwater. This can lead to reduced biological oxygen demand (BOD) and eventual suffocation of marine species; algae blooms can cover aquatic ecosystem feeding on the input of nutrients; fisheries and shellfish beds can be closed due to toxicity (EPA 2011). The waste water that enters the environment through CSO has a direct negative impact on the local environment. To compound this, increasing urbanization and waste water as well as increasing extreme rainfall events are making CSO events more frequent (Semadeni-Davies et al., 2008). By reducing the amount of water that spills out of CSOs during extreme rainfall events, the toxins and chemicals in waste water can be redirected to a waste treatment plant if there is one available. For developing cities that continue to empty raw sewage into the environment, there is little benefit to reducing CSOs before implementing waste water treatment plants.

There can be long term implications and consequences for continued CSO events. Under the European Union Water Framework Directive there is a list of priority pollutants to remove from waterways. In Paris, Gasperi et al. (2008) analyzed 66 of these priority pollutants finding 33 in raw sewage and 40 in wet weather effluent from CSOs. Urban runoff brought pollutants to the River Seine farther downstream than raw sewage. By allowing for the combination of sewage and urban runoff in CSO, urban waterbodies receive a toxic mix of chemicals. Under systems such as those set forth by the EU, there are legal consequences for these breaches in chemicals.

## 7 Limitations to policy

Policy needs to be created formally through the city government. Despite the clear co-benefits (see previous section), there can be barriers when creating policy; both from within and from outside the governmental system. These barriers hinder both the formation of new policy as well as the implementation of existing policy. It is important to understand these limitations in order to formulate more effective policy in the future. This discussion of limitations to policy is organized under the following headings: Political, Economic, Social and Environmental. The main limitations are summarized in Table 5 using the interviews from Table 3.

**Table 5:** Summary of limitations faced by cities when forming or implementing policy that reduces urban plastic leakages.

Political	Economic	Social	Environmental
Lack of support from regional and national governments	Recycling costs of cheap materials	Public responsibility and passing the torch	Uncertainty of rainfall projections
Lack of support from the international and transnational level	Cost of changing infrastructure	Lack of practical backing on convictions and fines	Storm surges and inundation
Making policy beyond the aesthetics	Perceived cost of damages	Active engagement of society over knowledge	
Power of lobbying	Funding for cleanup campaigns	Does society care	
Lack of legal backing to policy targets			
Working within regional and national plans			
Size of the city			

The four cities represented; Vancouver, Sydney, Miami Beach and New York City all have different demographics, environment and political management; as represented during the interviews. However, the cities were mostly in agreement when it came to the limitations faced by policy in reducing urban plastic leakages to the environment (Table 6). All five interviewees agreed that policy on street litter needs support from higher governments; yet public knowledge of the issue is high and public engagement is equally high in areas of direct control. Only the interviewee from Sydney suggested that they do have some legal backing to their policy targets through the NSW state government while the others all admitted to low legal backing. Furthermore, all the interviewees agree that aesthetics are a high motivation for policy formation while only New York City suggests the environment plays an equally important role in this process. New York City also differs from Miami Beach and Sydney by suggesting this problem is of a high priority for the city. While each of these cities is different, there are clear levels of agreement and disagreement to the extent the limitations effect their legislation.

**Table 6:** Summary of the agreements and disagreements between the interviewees on the limitations of policy in Vancouver, Sydney, Miami Beach and New York City.

	Vancouver A	Vancouver B	Sydney	Miami	NYC
Legal backing of policy targets	Low	Low	Moderate	Low	Low
Support from higher governments	Needs better integration	Needs better integration	Needs better integration	Needs better integration	Needs better integration
Public knowledge	High public knowledge	High public knowledge	High public knowledge	High public knowledge	High public knowledge
Public engagement	High in areas of direct control	High in areas of direct control	High in areas of direct control	High in areas of direct control	High in areas of direct control
Legal backing of laws and fines	Good with room for improvement	Good with room for improvement	Good with room for improvement	Little practical backing	Little practical backing
Motivation for policy	Aesthetics	Aesthetics	Aesthetics	Aesthetics	Aesthetics and environment
Priority for cities to tackle plastics	N/A	N/A	Low	Low	High

### 7.1 Political

Within the political system there are large barriers towards forming policy to prevent plastic leakages. Often, there is little support from the regional or national government. All five interviewees state that the existing policy is mostly driven by the cities themselves. Miami Interviewee (2016) states “we see zero initiative from the state of Florida and only guidelines from the Federal Government but these are local issues really.” While the immediate effects of plastic leakages are a local issue, without a higher government's support a city can struggle securing the necessary funding for programmes as well as obtaining the legal power to make changes. Vancouver Interviewee A (2016) states “we don't necessarily have the authority that would allow us to outright ban materials from use” and Sydney Interviewee (2016) agrees with “clearly we need support and a bit more legislative control on the use, production and disposal of plastics.” As the authority to make laws and bans on plastic has not been distributed to the city alongside the city lacking the support from higher governments, there is a limitation in the ability to generate impactful legislation. While some cities have passed bans, NYC Interviewee (2016) reflects on New York State's unwillingness to accept previous legislation New York City has made such as taxes on plastic bags and bans on Styrofoam food containers. These challenges are not as influential in more micro-state cities such as Singapore where the city government is more strongly tied with the national government, but the majority of cities suffer from this lack of legislative control.

Cities have to interact with regional policies; which has both benefits and limitations. Citizens can be confused between the role of the city and the state in areas such as waste management (Vancouver Interviewee B, 2016). While all interviewees agree that there is generally little confusion for citizens between city departments, the role of state departments adds uncertainty therefore leading to mismanaged waste or incorrect procedures in disposing of plastic. However, state policies can often feed into city policies thus helping cities achieve their goals. Sydney Interviewee (2016) describes how “ten years ago the government of NSW invested about AUS$80 million [~ US$61 million] on stormwater catchment planning.” From this regional investment, Sydney's councils were able to secure funding for infrastructure, education and management plans. While regional governments can severely limit city policy, these governments can play a helpful role when their goals are aligned with the city.

City governments often face pressure from outside the government to formulate policy. The plastics industry has a direct stake in this policy and therefore is a powerful lobbying group when the legislation is drafted (NYC Interviewee, 2016). While the plastics industry is not necessarily an antagonist in preventing plastic leakages, legislation that targets the industry instead of citizens receives more scrutiny from this powerful lobby. Other key industries can influence the decision-making process. In Miami Beach, the cruise line industry and boating industries are a large component of the marine economy (Miami Interviewee, 2016). While they set their own policy for plastic management they also have a stake in the policy set by Miami Beach in how to manage the waste. In addition to industry, citizens and city beautification is a driving role in formulating policy. All five interviewees agree that aesthetics has a major role in our decision making against plastics. Vancouver Interviewee A (2016) states “I think by dealing with the aesthetics we're also dealing with the environmental issues. People are concerned by the environmental issues but it's what they see every day that motivates the changes.” Cities want to have a clean, beautiful environment. However, a beautiful environment does not mean the problem is necessarily solved, rather it's out of sight. Citizen's desire for beauty mixed with industry's desire for non-limiting legislation politically hinders a city's ability to produce strict legislation on the control of plastics.

A large political limitation to policy is that, once the comprehensive plans are passed, the goals set forward are not legally binding. This limits the accountability for cities if they fail to reduce plastic leakages. NYC Interviewee (2016) states:

“It's great to set goals and milestones you want to hit but again its surface level. [Policy makers] don't explain how they're going to do things, what this is going to look like. Let's reduce X by Y amount but [policy makers] don't explain further. And really I think a lot of people don't ask those questions because once you go to the average person they're usually pretty excited that progress is being made on that front but they don't really have the knowledge or the background or the experience of working with the elected officials and city council to understand that… [Policy makers] also know that by the time anything is revealed, they're going to be gone. It transfers the responsibility.”

Without legal backing to the policy, the city is under no pressure to adhere to their targets. Once the targets come into place in 2025, 2030 or 2050 for example, the current policy makers are likely to be out of office thus passing the responsibility of the targets to future generations. This is compounded by the citizens' surface level understanding of how the policy targets will be met. The policy targets for plastic leakage reduction are often more statements of public relations than clear cut goals.

The city is not always limited politically to create legislation for plastics. Often targets would be impractical for a city to implement. It is difficult to permit and license for stormwater as it's difficult to control its quantity and quality (Sydney Interviewee, 2016). However, other controls on CSO and land use try to overcome this challenge. Furthermore, cities often try and work within the context of a greater regional strategy. Vancouver works closely with the regional plans set forward by British Columbia (Vancouver Interviewee A, 2016). Despite all the difficulties in implementing policy in cities, the large global cities are often used as an example for their regions, nations and globally (NYC Interviewee, 2016). By passing legislation in these cities of global importance, policy has the chance to spread throughout the world and different levels of government.

### 7.2 Economic

Policy that protects urban coastal waters from plastic leakages is not only limited by the political structure but also the cost. Not every plastic material is easily recyclable which inhibits the motivation to develop comprehensive recycling schemes. Despite their presence, “it costs a lot of money to collect lightweight materials that are worth very little” (Sydney Interviewee, 2016). Materials like polystyrene, while recyclable, do not produce an end product that is worth the cost of collection and processing. Therefore, for cities that are frequently strapped for funding and with priorities larger than the plastic problem, these materials are often excluded from deposit schemes discouraging recycling efforts. However, councils do “invest millions of dollars in waste collection and reuse and treatment. And also looking at things like street sweeping… which again cost councils multi-millions per year” (Sydney Interviewee, 2016). Cities do try to manage waste, but the financial cost of management limits further policy.

The cost of upgrading existing infrastructure often limits a city's ability to further push through legislation on the control of plastic on the streets. End of pipe solutions for stormwater and waste management facilities are a costly investment that takes years of planning and millions in funding to operate (Vancouver Interviewee A, 2016). In addition, they have a clear end of life and costly maintenance. Instead of creating new infrastructure, cities have opted to spend money on overhauling existing infrastructure to make it as efficient and environmentally friendly as possible (Sydney Interviewee, 2016). While creating a new sewer system would be the best environmentally, cities are now focusing on greening their landscape and working within current infrastructure as it is the most cost friendly. While this is not entirely negative due to the benefits greenspaces bring to a city, it demonstrates the power of economics on influencing the decision-making process. Furthermore, large infrastructure projects take time and most cities don't have the time to wait on these developments (NYC Interviewee, 2016); green infrastructure offers a faster solution and less money spent on work and disruptions. However, green infrastructure needs to be managed and this can add costs to both cleaning green infrastructure of trapped plastic debris as well as landscaping and maintaining the infrastructure. Cost and time largely direct the types of policies cities pursue in reducing plastic spillages.

Uncertainty in the future creates an economic doubt on the influence of policy. With a fast-changing climate and fluid demographics it's difficult to plan beyond the 20–50 year targets current policy outlines. Therefore, plans and programmes that continue into the years beyond are questioned because it may be unnecessary to invest for a future that won't appear in reality. While this reasoning can be logical for economic decisions, the climate models predict future extreme rainfall events are likely to be more severe, and therefore the damages brought with them are also likely to get more severe. If a system isn't put into place for the potentially destructive future, economic losses will be higher than they are today. Cities work economically in a more practical than precautionary way. In this they'll gratefully partner with NGO and non-profit organizations for education campaigns and cleanup campaigns but will often neglect funding regular cleanup activities themselves (NYC Interviewee, 2016). It is easier for cities to rely and sponsor smaller organizations than create one and fund one themselves. Cities often decide that the cheaper option is better than the more expensive, especially in areas that are considered low priority such as marine plastics.

### 7.3 Social

Policy will ultimately not be effective if the public do not take responsibility for their share of solving the plastic leakage problem. All five interviewees agreed that cities try to pass the torch of responsibility onto citizens because “we can't be everywhere; we can't do everything” (Vancouver Interviewee A, 2016). By engaging the public, cities can compensate for their lack of funding and ability to monitor all plastic usage. Furthermore, engaging citizens changes the way plastic interacts in the city on a day to day basis aiming for a reduction in plastic that ends on the street as litter. However, it is difficult “to create blanket legislation and expect everyone to be ok with it” (NYC Interviewee, 2016). Cities are a diverse place and no one legislation is going to be able to benefit all citizens. In addition to trying to get citizens to engage with policy, cities attempt to get industry to engage. Cities hope “that industry is going to step up and take… a bit more environmental responsibility… with providing the community with different alternatives” (Sydney Interviewee, 2016). Engaging industry is critical when cities attempt to ban and tax plastic materials. Yet developing policy that is both pleasing to policy makers, citizens and industry often limits the strength of the policy if these stakeholders are not engaged with the issue.

Cities hope to rely on social policing to alter consumer behavior with plastic. People are more likely to clean their litter, recycle their water bottles, reuse a plastic bag, etc. if they believe it to be the social norm and that their peers are observing and potentially judging their behavior (NYC Interviewee, 2016). However, there are always people in a community that don't follow these rules whether with ignorance or defiance (Miami Beach Interviewee, 2016). These people can get away with disregarding the norm as there is no “plastic police” (Sydney Interviewee, 2016). The interviewees varied on the extent they believe their city can handle littering fines and convictions, with Vancouver being the strongest. However, the three other interviewees admitted to never witnessing a fine being issued while frequently witnessing littering. While having a fine in place is a good policy, without proper enforcement it becomes societies' decision on their own self enforcement and currently there is a section of society in most cities that will actively or passively ignore anti-littering campaigns. It is a reason why cigarette butt littering remains a high priority for cities despite increasing access to butt disposals and campaigns to quit smoking.

Beyond informing the city about legislation, the city struggles to turn that knowledge into active engagement. Cities have higher response rates in areas where they have direct control, such as for example single family homes (Vancouver Interviewee A, 2016, Vancouver Interviewee B, 2016). Enforcing policy becomes more difficult in areas where the city government interacts with landlords and property managers and not the citizens using the space. After informing citizens of a new policy, cities experience better response rates among citizens that have more frequent direct contact with city institutions as opposed to those that go through land lords or estate managers. Large global cities also deal with changing population. Beach tourists may actually protect the environment better than locals because of increased scrutiny on their actions or their less frequent interaction with daily plastic i.e. most meals consumed in restaurants so less plastic taken on the street (Miami Beach Interviewee, 2016). Locals often suffer from a lack of self-awareness and sense of place. Large cities have transient populations and people may not identify with their current place of residence as being home. This is compounded when cultural and linguistic differences emerge. Furthermore, policy differs from city to city making it difficult for these transient populations to relearn habits. Active engagement with cities is difficult when knowledge is itself difficult to spread.

In daily life, citizens may be ignorant to the problem of coastal marine plastic. Many people in coastal cities don't venture out to the beach and witness the problem first hand (NYC Interviewee, 2016). If one does not see the problem regularly, it is easy in daily life to disconnect a discarded bottle on a street from a discarded bottle harming sea life. A city's environment is interconnected but an individual's life may not encompass the entire environment, making it difficult to create a sense of urgency for problems unseen.

Finally, a large social limit to policy is the citizens' commitment to the environment in their day-to-day lives. Plastic bag charges are good at reducing plastic bag usage, but over time citizens may incorporate the tax into their expected spending and that could cause a rebound of plastic bag use (Vancouver Interviewee A, 2016). Homeless populations can increase street litter by emptying bins searching for items with deposits on them (Vancouver Interviewee A, 2016). Citizens act largely on economic concerns. There is always a fraction of society that campaigns heavily for the environment but the majority of citizens respond to economic incentives. Therefore, despite all the education and cleanup campaigns, there may not be success without the encouragement of financial incentives or politically enforced penalties. While social policy may be viewed as a solution for policy makers to alter peoples' behavior in using plastic, peoples' behavior may be the strongest limitation to the very policy itself.

While the environment may not directly limit policy making, the future environment does influence our decision making. The rainfall projections are not completely certain, they are not fact. While models can project the likelihood of whether rainfall increases or decreases, these models will always have an uncertainty range when quantifying the increase in rainfall or frequency of storms. Therefore, cities may be over or under preparing themselves for future rainfall. Coastal cities are not only susceptible to rainfall, but also coastal flooding. Coastal flooding, sea level rise and storm surges can flush cities of street plastic and create further debris if they are destructive. Furthermore, rainfall changes are only one consequence of future climate change. Cities must also prepare for changes in drought, hotter or colder temperatures and other changes to the physical system.

Within the city, environmentalism runs throughout city issues. It is often the poorest citizens that live in the least environmentally secure areas (NYC Interviewee, 2016). Yet all citizens can enjoy the coast and the beaches and every citizen uses waste and water management in some form. Rainfall projections and increased storms are not a standalone issue for cities and all citizens will be influenced by future environmental changes and policy initiatives in the future. Policy protecting the oceans from plastics is limited by political, economic and social factors. While one limitation alone may not be enough to stop policy when these factors merge together policy may neglect the care of the urban waterways.

## 8 Recommendations for policy makers and conclusions

**Fig. 3:**
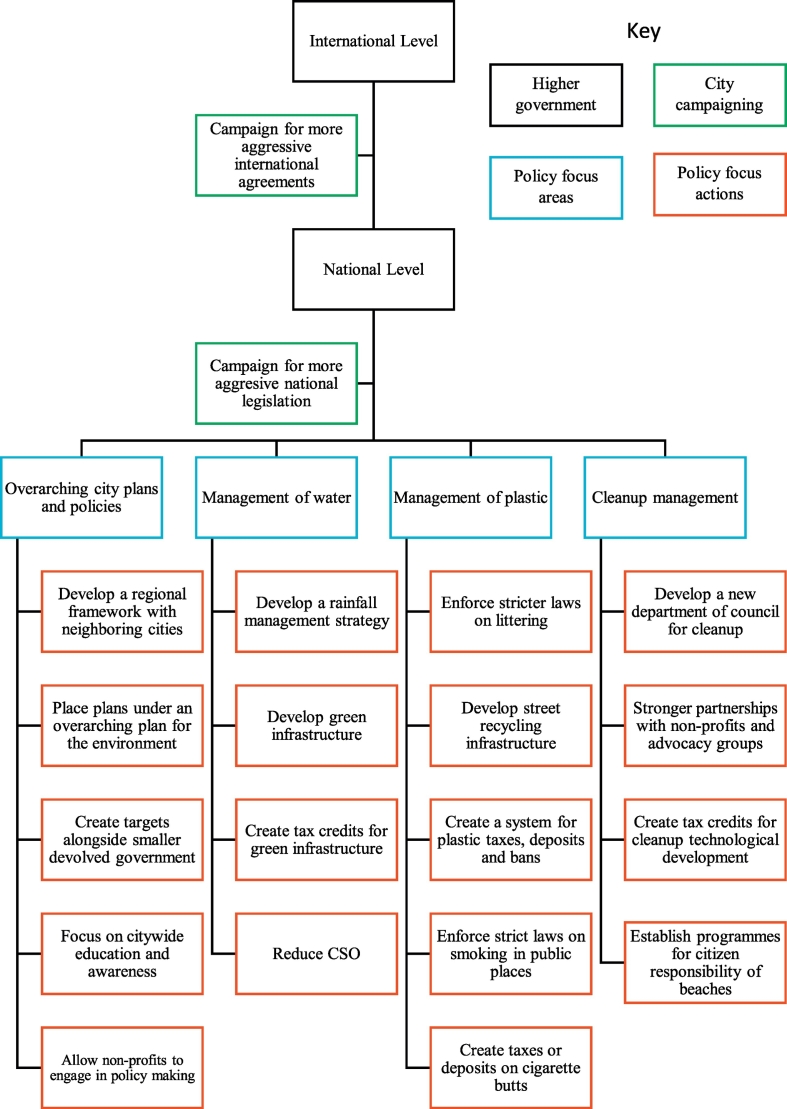
Suggestions and intervention points for cities and policy makers to create and enforce policies that reduce plastic leakages to the marine environment.

Policy that targets urban plastic leakages to the ocean is highly beneficial for a city and its environment; yet the political, economic, social and environmental barriers often hinder the extent this policy to tackle litter is implemented. This paper attempts to put forward suggestions for coastal cities in how to create future policy that overcomes the limitations in the context of existing legislation. Cities should continue to advocate for both stronger international and national laws and agreements on marine plastics (Fig. 3), however, the following suggestions are targeted at a city-level approach to solving the problem. The suggestions are organized into four categories (Fig. 3): overarching city plans and policies, management of the water, management of the plastic waste, and cleanup management. While each city is structured differently, these suggestions are geared for large international coastal cities and their surrounding communities with direct interactions with the ocean. Inland cities may find these recommendations useful as they touch on the underlying theme of urban environmental management.

### 8.1 Overarching city plans and policies

• To overcome weak regional and national level support for laws targeting the reduction of plastic usage, cities can create a regional committee representing the city and neighboring communities to create policy. In this the entire metropolitan region is formulating a single policy and citizens living in the entire region follow the same criteria whether it be bans, fines, taxes or deposits.
• To overcome weak targets set in policy, cities should place their forward-looking plans under an overarching plan for the environment and sustainability. Thereby, each individual strategy for example for waste management or water management is placed in the context of the general city's environmental goals. It also allows for the strategies to be specific and targeted to areas of concern while working towards a common goal for the city. This also encourages different city departments to work closer together to achieve these goals closing any gaps in management.
• To overcome other weak targets set in policy, cities should create targets alongside the community. City governments can work towards 50% of the target while the remaining 50% is left up to communities. For example, in increasing recycling rates, the city can increase recycling infrastructure and alter plastic collection methods while districts within the city are responsible to reduce plastic waste being disposed of improperly. In this the city is more likely to meet their half of the target as they are not required to do everything towards the goal. Furthermore, the city districts can develop strategies tailored to their specific community to reduce mismanaged waste.
• To overcome citizens' lack of engagement with the plastics problem, cities should create and further education and awareness campaigns. These campaigns can target public areas where citizens are most likely to interact with the campaign such as public transit stations as well as schools which can help shape future citizens' behavior. The increased presence of these campaigns can slowly permeate daily life and decisions.
• To overcome citizens' lack of engagement with the plastic problem, cities should allow for non-profit advocacy to engage in the policy formation stages. The non-profit advocacy groups have specific knowledge in more niche fields and their practical knowledge is valuable. Furthermore, these groups have experience with public engagement in taking action against marine plastics and have valuable experience in the application of policy. As opposed to lobbying the NGOs should be present during the drafting of legislation as contributors in contrast to persuading legislators.

### 8.2 Management of the water

• To better control extreme rainfall events, cities should develop a rainfall management plan that examines how rainfall collects over the city, its current effects on the environment and the effects of future rainfall projections. The city will be better prepared to respond to extreme rainfall events, have a better understanding of how rainfall interacts in the city and understand how plastic is moved through the inundated environment.
• To better control extreme rainfall events and capture street litter, cities should focus on developing green infrastructure. The infrastructure is often cheaper than traditional methods, can occupy both large and small spaces, and has numerous benefits towards the health and environment of the city. Green infrastructure may also be cheaper to maintain and is easier to change in the future. This infrastructure will also help manage water and plastic during normal dry and wet events. Funds should also be set aside to maintain and clean the infrastructure.
• To better develop green infrastructure, cities should establish a tax credit system for property owners who invest in developing green infrastructure. As the base cost of introducing green infrastructure projects may not be as large to cities, depevolment of green infrastructure can be unattainable for small businesses and home owners without a financial incentive. The tax credit can be dictated by density zones, with the densest areas receiving the biggest credit.
• To better control plastic leakages to the marine environment, cities should focus efforts on reducing Combined Sewer Overflows (CSO). While reducing the flow of water to CSO can be done with green infrastructure, closing the CSO will ultimately prevent sewage from entering the waterways. The infrastructure improvements should also reroute stormwater to water treatment plants as opposed to direct spillage into the environment.

### 8.3 Management of the plastic

• To better control the use of plastic in the city's streets, cities should create strict laws and steep fines on littering. These laws and fines should be monitored closely immediately after implementation and strictly enforced to encourage citizens to change habits. Once the new habits take hold, enforcement can be scaled back with increased social policing.
• To better control the use of plastic in the city's streets, cities can incorporate better street recycling vessels. These include recycling containers within proximity of waste bins, separate containers for different materials including plastic, separate containers for items with deposits, or bins that can accept deposited materials and return the value of the deposit to the user. The convenience of disposal of plastics will encourage citizens to dispose of plastic correctly on the streets.
• To better control the overall use of plastic, cities should identify necessary and unnecessary plastic products for daily life. Afterwards, the city should apply a tax or deposit on their ‘necessary’ plastic products such as plastic bags and bans on their ‘unnecessary’ products such as Styrofoam cups. The lists should be tailored to each city as for example plastic bags may be viewed as necessary in one but unnecessary in another depending on the residents. Environmentally friendly cities are becoming more enticing for investments. Cities push each other towards higher environmental standards to maintain a competitive edge.
• To better control cigarette butts in cities, cities should create more community areas where smoking is not permitted. Public parks, beaches, and commercial areas should be areas of priority for cities seeking to reduce both cigarette butts and second hand smoke hazards.
• To better control cigarette butts in cities, cities can implement a deposit or further tax on the cigarettes. The tax can offset costs of collecting cigarette butts in the street while a deposit encourages smokers to not dispose of their waste on the street.

### 8.4 Cleanup management

• To better cleanup the coastline and local waterways, cities should create a new department or sub-department/committee that is dedicated towards marine and coastal cleanup. By doing this, cities would be able to create a direct strategy for cleanup and allocate specific resources to this. It will also be able to direct citizens concern and involvement towards a specific department.
• To better cleanup the coastline and local waterways, cities should partner with non-profits for better environmental management. In the actual cleanup, cities should subcontract and fund the responsibility to these non-profits who can dedicate their time to the specific problem of marine plastics.
• To better cleanup the coastline and local waterways, cities should establish tax credits for companies and start-ups pursuing technologies that clean up the debris. This will encourage citizen engagement with clean-up as well as reduce the burden to the city governments to search for solutions.
• To better cleanup the coastline and local waterways, cities should establish programmes that allow for citizens or businesses to take ownership of the beaches such as adopt-a-beach programmes. This will further encourage citizen engagement with the environment and reduce the cleanup costs to cities.

Most cities have policy in place that control plastic leakages. There is no standard in management, however, and often the policies are conceptual in nature and lack legal follow up in the city and regional governments. Reducing urban plastic leakages can have numerous benefits to the city beyond solving the plastic problem. The city can experience a stronger economy, a cleaner environment and a healthier society while removing plastic leakages. While cities struggle to politically formulate policy or find the appropriate funding for new measures, a lack of social engagement will ultimately limit a policy's effectiveness.

By implementing a selection of the recommendations, this paper believes that cities can effectively overcome the limitations towards policy targeting plastic and enjoy the direct and co-benefits. However, each city is unique and this paper should be taken as a guiding document. Certain coastal cities continue to face a threat of extreme rainfall events and further research should be conducted on an individual city level to create comprehensive plans to reduce plastic leakages to the ocean during these events. Cities should continue to focus on reducing the amount of plastic waste produced. This way additional plastic is removed from the waterways and the management of the water systems and cleanup can be more effective in ensuring a greener environment.
